# Thickness-Tunable Bilayer PBAT Nanofibrous Scaffolds for Enhancing r-AdMSCs’ Tenogenic Commitment in Supraspinatus Tendon Regeneration

**DOI:** 10.3390/jfb17070310

**Published:** 2026-06-23

**Authors:** Serdar Onat Akbulut, Elvan Konuk Tokak, Tuğçe Gültan, Menemşe Gümüşderelioğlu

**Affiliations:** 1 Bioengineering Division, Graduate School of Science and Engineering, Hacettepe University, Ankara 06800, Turkey; onat_akbulut@hacettepe.edu.tr; 2 Department of Chemical Engineering, Faculty of Engineering, Hacettepe University, Ankara 06800, Turkey; elvankonuk2368@gmail.com (E.K.T.); gultantugce@gmail.com (T.G.)

**Keywords:** Poly(butylene adipate-co-terephthalate) (PBAT), electrospinning, nanofibrous matrices, r-AdMSCs, supraspinatus tendon, tenogenic differentiation

## Abstract

Acute or chronic rotator cuff tears are major causes of shoulder dysfunction, motivating the development of scaffolds with tailored thickness and mechanics for supraspinatus tendon regeneration. This study aimed to investigate the effect of bilayer poly(butylene adipate-co-terephthalate) (PBAT) scaffold thickness on the tenogenic differentiation of rat adipose mesenchymal stem cells (r-AdMSCs) and supraspinatus tendon regeneration. Aligned fibers with a diameter of approximately 476 nm were deposited onto randomly oriented layers at different times (4 h; 4S, 6 h; 6S, 8 h; 8S), and scaffolds with increasing thicknesses from 441 µm (4S) to 1132 µm (8S) were produced. Mechanical testing showed comparable tensile strength for 4S and 6S (≈1.9–2.0 MPa) and modulus (5.5–7.3 MPa), while 8S exhibited markedly reduced stiffness (0.5 MPa) and hyper elastic deformation. Mechanical performance across degradation conditions remained strongly thickness-dependent: thinner scaffolds retained integrity and strengthened, with modulus increases during hydrolytic and enzymatic degradation, whereas thicker matrices showed limited remodeling and instability. Rat-AdMSCs’ were cultured on the scaffolds for 21 days. Cell-free and cell-laden mechanical responses further reflected thickness effects: cell-free samples stiffened due to media-induced passive matrix tightening, whereas cell-laden scaffolds showed extracellular matrix (ECM)-driven reinforcement, most prominently in 4S, which reached 2.1 MPa tensile strength with improved elasticity and balanced deformation. The 4S scaffold exhibited the highest tensile strength and significantly increased collagen-1 (col1), tenomodulin (tnmd) and scleraxis (scx) expression compared with the other groups. In conclusion, among all groups, 4S scaffolds demonstrated the most favorable mechanical and biological performance, suggesting that scaffold thickness plays a critical role in regulating tendon regeneration and will become even more suitable when matured in bioreactors.

## 1. Introduction

The supraspinatus (SSP) and infraspinatus tendons, both integral components of the rotator cuff tendon group located in the posterior region of the shoulder, play a vital role in facilitating shoulder joint movement and maintaining joint stability [1]. Full-thickness rotator cuff tendon tears, whether acute or chronic, are frequently encountered in clinical settings and represent a major cause of shoulder dysfunction. Among these, SSP tendon tears are particularly prevalent, often resulting from age-related degeneration, repetitive overuse, or traumatic injury [2].

The treatment of SSP depends on tear severity, ranging from conservative approaches—such as physical therapy, activity modification, medication, injections, and other modalities—to surgery in more severe cases. Healing without surgery may take several months. Arthroscopic repair is the gold standard, involving removal of inflamed tissue and suturing the remaining tendon to bone. However, full functional recovery is not always achieved, and the tears may recur after further trauma [3].

The SSP tendon comprises two morphologically and anatomically distinct regions: anterior and posterior. The anterior region is thicker and cylindrical, with surrounding fibrous tissues functioning synergistically with the SSP muscle. In contrast, the posterior region is thinner and planar and operates largely independently of the muscle [4]. The SSP tendon is subjected to complex loading conditions and exhibits pronounced region-specific characteristics, particularly in the posterior region, where increased mechanical demand, limited vascularization, and distinct elastic behavior coexist [5,6].

Biomechanically, the elastic modulus of the anterior region (~600 MPa) is substantially higher than that of the posterior region (~200 MPa), indicating that the anterior region is stiffer and, consequently, exhibits lower resistance to tearing. Conversely, the posterior region demonstrates greater flexibility due to its higher elongation capacity. Owing to its compliant nature, tears in the posterior region are more frequently associated with chronic overuse and mechanical fatigue. Despite its susceptibility to injury, the posterior region exhibits a greater regenerative potential than the anterior region, attributable to its enhanced capacity for cellular infiltration. Approximately 47% of the anterior region consists of tendon tissue, whereas this proportion increases to nearly 70% in the posterior region. This structural distinction suggests that mechanical loads in the anterior region are predominantly transmitted through muscle tissue, whereas in the posterior region, the majority of load is borne directly by the tendon. These marked structural and functional differences render the posterior region a particularly relevant yet underexplored model for investigating rotator cuff tendon injuries [5,6,7].

Tissue engineering strategies for SSP tendon regeneration aim to replicate the native viscoelastic and elastic properties that enable energy storage, recoil, and precise force transmission [7]. Accordingly, scaffolds designed for posterior SSP tendon repair must combine sufficient mechanical strength, elasticity, and a degradation profile that aligns with the natural healing timeline of the tissue [8].

Although both natural and synthetic polymers have been extensively investigated in rotator cuff tissue engineering, each class presents inherent limitations. Natural polymers generally lack the mechanical robustness required for load-bearing applications, whereas synthetic polymers offer superior durability but comparatively limited bioactivity [8,9]. Biodegradable synthetic polymers such as poly(lactic acid) (PLA), poly(glycolic acid) (PGA), and poly(lactic-co-glycolic acid) (PLGA) have been widely employed in SSP tendon repair in woven, nonwoven, and electrospun patch formats. Although these materials offer tunable mechanical properties and controlled degradation, they tend to lose mechanical support over time and still fall short of replicating the region-specific mechanical organization of the SSP tendon [9,10]. Similarly, non-absorbable synthetic meshes such as polytetrafluoroethylene (PTFE) offer high initial mechanical stability but suffer from poor biointegration and chronic foreign body reactions, limiting their long-term clinical applicability [9]. Natural polymers, including collagen, gelatin, chitosan, hyaluronic acid, and peptide-based hydrogels, promote cellular adhesion and biological signaling but lack sufficient mechanical strength for load-bearing tendons such as the SSP. Consequently, these materials are typically incorporated into hybrid systems in combination with synthetic polymers [11,12].

Among the fabrication strategies explored in tendon tissue engineering are electrospinning, which enables the generation of highly aligned fibrous architectures that closely mimic the native extracellular matrix (ECM); 3D printing and other additive manufacturing techniques, which allow precise control over scaffold geometry and spatial organization; and conventional methods such as solvent casting, phase separation, and textile-based techniques—including braiding and weaving—which are particularly well suited for producing mechanically robust constructs. Each of these approaches presents distinct advantages and limitations with respect to key parameters essential for functional tendon regeneration, namely fiber alignment, porosity, scalability, and mechanical performance [9,10,11,12].

Electrospun nanofibrous scaffolds represent a significant advancement in SSP tendon tissue engineering, as their fiber diameters and alignment closely mimic the hierarchical organization of native tendon collagen. Aligned nanofiber mats have been shown to promote cell alignment and tendon-like ECM deposition. However, many studies have focused primarily on fiber alignment, with limited consideration of thickness-dependent or region-specific mechanical heterogeneity, particularly in the posterior region of the SSP tendon [12,13,14].

To overcome these limitations, bilayer and multiphase scaffold designs have been proposed [15,16]. The bilayer structure not only provides mechanical support but also facilitates mass transport, cell filtration, and cell–matrix interactions. Nevertheless, most existing bilayer systems have been developed to target the tendon–bone interface or to spatially segregate biological and pharmacological functions, such as growth factor delivery, drug release, or adhesion modulation. Consequently, bilayer constructs in rotator cuff repair have largely been implemented as interface-oriented patches or microenvironment-regulating platforms, rather than as systems designed to replicate the intrinsic regional mechanical organization of the SSP tendon itself, particularly its posterior region [15,16].

Cell sheet engineering has emerged as a promising strategy for recreating tendon anisotropy in scaffold-free and hybrid scaffold-based constructs. Using thermoresponsive PIPAAm-grafted substrates, aligned cell sheets can be generated while preserving cell–cell interactions and ECM organization, enabling the formation of 3D tissues with controlled cellular orientation. These anisotropic sheets promote the deposition of aligned collagen fibers that resemble the native tendon architecture [17]. Furthermore, hybrid systems combining MSC sheets with growth factor-loaded biomaterials and supportive scaffolds have demonstrated enhanced tenogenic differentiation, collagen organization, and biomechanical properties, highlighting the potential of anisotropic cell-sheet technologies for tendon tissue engineering [18].

In contrast, the present study introduces a bilayer scaffold concept that is independent of the tendon–bone interface and specifically targets the posterior region of the SSP tendon. The scaffold is composed of two distinct fiber architectures derived from the same polymer, rather than from different materials. An aligned fiber layer promotes cellular orientation and tendon-like structural organization, while an underlying randomly oriented fiber layer enhances porosity and capillary-driven transport, facilitating the diffusion of nutrients, oxygen, and soluble factors. Rather than serving merely as passive mechanical support, the random fiber layer functions as a vessel-like architecture that supplies the aligned layer, thereby mimicking the limited vascularization characteristic of the posterior SSP tendon. By integrating aligned and random fiber architectures within a single polymer system, mechanical and chemical continuity between layers is preserved, minimizing interfacial incompatibilities commonly associated with multi-material scaffold designs.

Within this framework, poly(butylene adipate-co-terephthalate) (PBAT), although not previously reported in tendon tissue engineering applications, was identified as a promising synthetic candidate due to its flexibility, high elongation capacity, and processability with electrospinning. Polymers such as PLA and PGA can exhibit limitations in maintaining fiber morphology due to their low melting temperature and slow crystallization kinetics; in contrast, PBAT, owing to its aromatic–aliphatic backbone, integrates enhanced thermal stability with chain flexibility, enabling improved processability and more effective replication of the anisotropic architecture of the SSP tendon [19,20,21,22]. Accordingly, a two-layered anisotropic nanofibrous PBAT scaffold was developed as a model system for the posterior region of the SSP tendon. We hypothesized that rat-derived adipose mesenchymal stem cells (r-AdMSCs) undergo tenogenic commitment when cultured on bilayer anisotropic thickness-dependent PBAT scaffolds characterized by a high elastic modulus and reduced thickness. To test this hypothesis, nanofibrous PBAT matrices with a randomly oriented base layer were fabricated via electrospinning, followed by deposition of an aligned PBAT fiber layer to form a bilayer construct. This study is designed as an intra-material optimization of process (electrospinning duration/matrix thickness) and does not aim to compare PBAT with other already reported polymers and one-layered scaffolds. The mechanical properties of the scaffolds were systematically evaluated to assess structural integrity and stability. Subsequently, in vitro cell culture studies were conducted to investigate the tenogenic differentiation potential of r-AdMSCs cultured on the engineered bilayered thickness-tunable PBAT scaffolds.

## 2. Materials and Methods

### 2.1. Materials

Poly(butylene adipate-co-terephthalate) (PBAT; EcoFlex^®^) was purchased from BASF (Kocaeli, Turkey). 1,1,1,3,3,3-Hexafluoro-2-propanol (HFIP), used as the solvent for electrospinning, along with sodium chloride (NaCl), potassium chloride (KCl), sodium phosphate dibasic (Na_2_HPO_4_), sodium azide (NaN_3_), lipase, and reagents for cell viability assays including Calcein AM, Ethidium Homodimer-1 (EthD-1), and 3-[4,5-dimethylthiazol-2-yl]-2,5-diphenyltetrazolium bromide (MTT), were all obtained from Sigma-Aldrich, (St. Louis, MO, USA). Collagenase used in biodegradation studies was obtained from BioChrom (Berlin, Germany).

For cell culture, α-Minimum Essential Medium (α-MEM) was sourced from Multicell (Wisent Inc., Saint-Jean-Baptiste, QC, Canada). Fetal bovine serum (FBS), L-glutamine, penicillin/streptomycin (P/S), amphotericin B, and gentamicin were purchased from Cegrogen (Stadtallendorf, Germany). Dulbecco’s phosphate-buffered saline (DPBS) and trypsin/EDTA were obtained from Capricorn Scientific (Ebsdorfergrund, Germany).

For cytoskeletal and nuclear staining, bovine serum albumin (BSA; Capricorn Scientific, Ebsdorfergrund, Germany), Triton X-100 (Acros Organics, Geel, Belgium), glutaraldehyde (GA; 25% *v*/*v*, Sigma-Aldrich), Alexa Fluor^®^ 488-conjugated phalloidin (Cell Signaling Technology, Danvers, MA, USA), and 4′,6-diamidino-2-phenylindole (DAPI; Invitrogen, Carlsbad, CA, USA) were used.

For gene expression analysis, Trizol reagent (Qiagen, Hilden, Germany), chloroform (Merck, Darmstadt, Germany), an RNA isolation kit (Qiagen, Hilden, Germany), a cDNA synthesis kit (Applied Biosystems, Foster City, CA, USA), and EvaGreen^®^ dye (Solis BioDyne, Tartu, Estonia) were used for RT-qPCR.

For immunohistochemistry, sodium citrate buffer and Tween 80 (Sigma-Aldrich St. Louis, MO, USA), goat serum (GS, Capricorn Scientific, Ebsdorfergrund, Germany), anti-Scleraxis (Scx) antibody (Thermo Scientific, Waltham, MA, USA), anti-Tenomodulin (Tnmd) antibody (Bioss Inc., Woburn, MA, USA), and secondary antibodies Alexa Fluor^®^ 488 and Alexa Fluor^®^ 568 (Cell Signaling Technology, Danvers, MA, USA) were utilized.

### 2.2. Fabrication and Characterization of Bilayer PBAT Nanofibrous Scaffolds

In this study, bilayered PBAT scaffolds were fabricated to achieve the desired thickness while minimizing fabrication time. PBAT was dissolved in HFIP at a concentration of 12.5% (*w*/*v*) to prepare the electrospinning solution [23]. The solution was loaded into a syringe and processed using an electrospinning apparatus (Inovenso NE300, İstanbul, Turkey).

Initially, randomly oriented PBAT nanofibrous layers were fabricated via electrospinning onto static flat plates for durations of 4 h (4S), 6 h (6S), and 8 h (8S), respectively, to serve as the base layers. These matrices were subsequently dried overnight under a fume hood. Aligned PBAT fibers were then deposited directly onto each corresponding base layer for the same durations (4, 6, or 8 h), creating anisotropic bilayer scaffolds. Aligned nanofibers were deposited by rolling the base layer onto a rotating drum, which was then set to 2000 rpm.

**Figure 1:**
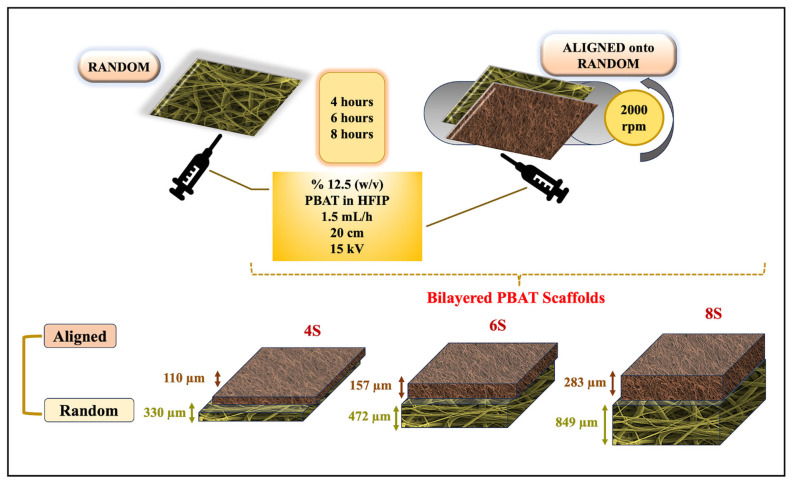
Schematic illustration of the fabrication process of bilayer nanofibrous PBAT scaffolds.

To ensure structural integrity and prevent delamination between layers, electrospinning parameters were kept constant throughout the process: a 20 cm needle-to-collector distance, a flow rate of 1.5 mL/h, an applied voltage of 15 kV, and a 15-gauge blunt-tip needle were used for both layers. These values were then systematically refined through preliminary optimization experiments (trial-error) to eliminate bead formation and achieve the desired fiber morphology [24]. The overall fabrication process is schematically illustrated in Figure 1.

Morphological characterization of the scaffold cross-sections and aligned surfaces was performed using scanning electron microscopy (SEM) (Tescan, FIB-SEM GAIA 3, Brno, Czech Republic). Fiber diameters were measured in ImageJ software (1.54J, NIH, Bethesda, MD, USA) by drawing lines perpendicular to the fiber axis. Fiber alignment was evaluated by determining the orientation angle of each fiber relative to a reference axis and normalizing all angles to 90°. Angular distribution histograms were generated from the normalized data, and fibers oriented within ±20° of the predominant direction were classified as aligned to calculate the alignment percentage.

The mechanical properties of the fabricated scaffolds were assessed using a uniaxial tensile testing device (TA.XTPlus, Stable Microsystems, Godalming, Surrey, UK). Prior to testing, samples (10 × 50 mm) were pre-incubated in PBS at 37 °C overnight to simulate physiological conditions. Tensile tests were conducted under a 50 N load at a constant crosshead speed of 10 mm/min, following the ASTM D638 standard [25]. During tensile testing, samples were mounted such that the aligned fibers were oriented parallel to the loading direction. Key mechanical parameters, including tensile strength, elastic modulus, and elongation at break, were derived from the stress–strain curves using the instrument’s accompanying software.

Biodegradation studies were performed over a 12-week period to simulate the timeline of tendon healing. Two types of degradation media were used: (1) enzymatic degradation medium composed of lipase (0.5% *w*/*v*) and collagenase (1.35% *w*/*v*, 5 U/mL) in PBS (pH: 7.4), and (2) enzyme-free PBS to serve as a hydrolytic degradation medium. Scaffold samples were fully immersed in the respective media, which were refreshed weekly to maintain enzyme activity and consistent degradation conditions. SEM analyses were performed on samples retrieved in weeks 4 and 12 to assess the impact of hydrolytic and enzymatic degradation on scaffold integrity. Tensile tests were applied for both groups in weeks 4 and 12 by following same protocol mentioned in this section.

### 2.3. Cell Culture Studies

Cell culture studies were conducted using rat-derived adipose mesenchymal stem cells (r-AdMSCs, passage 4). These cells were isolated and characterized as part of previous studies [26]. They were cultured in α-MEM supplemented with 10% (*v*/*v*) FBS, 1% (*v*/*v*) L-glutamine, 0.4% (*v*/*v*) P/S (100 units/mL P and 100 µg/mL S), 0.4% (*v*/*v*) amphotericin B, and 0.4% (*v*/*v*) gentamicin. The cultures were maintained in a CO_2_ incubator (Heraeus Instruments, Hanau, Germany) at 37 °C.

For seeding, cells were detached using trypsin-EDTA, centrifuged, and resuspended in complete medium. The 4S, 6S, and 8S PBAT scaffold samples were cut into 1 × 1 cm^2^ pieces for cell culture and 1 × 5 cm^2^ pieces for post-culture mechanical testing. Scaffolds were sterilized by UV irradiation for 1 h on each side. r-AdMSCs were seeded onto the aligned nanofiber surface at a density of 3 × 10^4^ cells/cm^2^. The cell culture was maintained for 21 days, with the culture medium refreshed every 3 days.

#### 2.3.1. Cell Viability and Proliferation Assays

Cell proliferation was evaluated using the MTT assay on days 7, 14, and 21 of the culture periods. For the MTT assay, the culture medium was removed, and 40 µL of MTT solution (2.5 mg/mL MTT powder dissolved in DPBS) was added to each well, followed by 400 µL of FBS-free culture medium. After a 3 h incubation at 37 °C, the MTT solution was removed, and 400 µL of DMSO was added to dissolve the formazan crystals. The resulting solution was transferred to a new 96-well plate (200 µL per well), and optical density was measured at 570 nm with a reference wavelength of 690 nm using a microplate reader (Asys Hightech Gmbh, UVM 340, Eugendorf, Austria) [27].

Cell viability was further evaluated using live/dead staining with Calcein AM and EthD-1 [28]. After removing the culture medium, the samples were washed three times with Ca^2+^ and Mg^2+^-supplemented DPBS (DPBS+). The samples were then incubated in DPBS+ containing 1 µM Calcein AM (for live cells) and 1 µM EthD-1 (for dead cells) at room temperature for 30 min. After incubation, the samples were washed three times with DPBS+ and visualized using fluorescence microscopy (Olympus Corporation, Tokyo, Japan). Live cells were stained green (Calcein AM), and dead cells were stained red (EthD-1).

#### 2.3.2. Cytoskeleton/Nucleus Staining

The cytoskeletal and nuclear morphology of r-AdMSCs cultured on 4S, 6S, and 8S samples were examined on days 1, 7, and 21 using Alexa Fluor^®^ 488 Phalloidin (Cell Signaling Technology, Danvers, MA, USA) and DAPI staining. After removing the culture medium, the samples were washed three times with DPBS and fixed by incubation in 2.5% (*v*/*v*) GA at room temperature for 30 min. Following fixation, the samples were washed twice with PBS and incubated in a PBS solution containing 0.1% (*v*/*v*) Triton X-100 for 10 min to permeabilize the cell membranes. The samples were then washed twice with PBS to remove any residual Triton X-100.

For staining, Alexa Fluor^®^ 488 Phalloidin (specific for F-actin) was prepared at a 1:100 dilution, and DAPI (for cell nuclei) was diluted 1:1000 in PBS/A (1% *w*/*v* BSA in PBS). The samples were incubated in the staining solution for 1 h at room temperature in the dark. After incubation, the samples were washed three times with PBS/A and visualized under fluorescence microscopy (Olympus, Japan).

#### 2.3.3. Reverse Transcription Quantitative Polymerase Chain Reaction (RT-qPCR) Analysis

Gene expression levels of Col1a1 (Col1), Col3a1 (Col3), Scx, Tnmd, and Tnc (tenascin C) were assessed by RT-qPCR [29]. On days 7 and 21 of culture, the samples were washed three times with DPBS and then dissected using microscissors. mRNA was isolated using the TRIzol method in combination with QIAzol Lysis Reagent and the RNeasy Mini Kit (Qiagen), following the manufacturer’s protocols. After isolation, the total mRNA concentration was quantified at 260 nm using a NanoDrop 2000 spectrophotometer (Thermo Scientific, Waltham, MA, USA). Complementary DNA (cDNA) was synthesized using the High-Capacity cDNA Reverse Transcription Kit on the SimpliAmp™ Thermal Cycler (Applied Biosystems, Foster City, CA, USA).

RT-qPCR was performed with the Viia7 Real-Time PCR System (Applied Biosystems, Foster City, CA, USA) using 5× HOT FIREPol^®^ EvaGreen^®^ qPCR Mix Plus. Gene expression data were normalized to the housekeeping gene β-actin, and fold changes in expression were calculated using the 2^−(ΔΔCt)^ method, comparing the samples cultured on 4S, 6S, and 8S scaffolds. The primer sequences used for each gene are listed in Table S1.

#### 2.3.4. Immunohistochemical Staining

Scx and Tnmd were assessed as specific tenogenic genotype markers using immunohistochemical staining. Samples were collected on days 7 and 21 of culture, washed three times with PBS, and fixed with 4% (*v*/*v*) PFA for 30 min at room temperature. Following fixation, samples were washed again with PBS.

Antigen retrieval was performed following the manufacturer’s protocol. Briefly, samples were placed in tissue cassettes and submerged in 500 mL of sodium citrate buffer (pH 6.0). The cassettes were then heated in a microwave oven at 850 W for 20 min. Immediately after heating, the samples were rapidly cooled in an ice bath and subsequently incubated in PBS containing 0.01% (*v*/*v*) Triton X-100 for 30 min at room temperature to permeabilize the membranes.

To block non-specific binding, samples were washed with PBS containing 5% (*v*/*v*) GS and then incubated in 5% GS for 1 h at room temperature. Following the blocking step, samples were incubated overnight at 4 °C with primary antibodies against Scx and Tnmd, each diluted 1:250 in PBS. The next day, samples were washed and incubated with appropriate secondary antibodies, Alexa Fluor^®^ 568 and Alexa Fluor^®^ 488, each at a 1:200 dilution.

Fluorescent staining was visualized using a fluorescence microscope (Olympus, Tokyo, Japan). Fluorescence intensities were quantified using ImageJ software. Images were converted to grayscale, background subtraction was performed, and mean fluorescence intensity values were calculated within predefined regions of interest. Measurements were conducted in triplicate for each experimental group.

#### 2.3.5. Post-Culture Mechanical Characterization

Mechanical testing was performed as described in Section 2.2 to evaluate the influence of ECM deposition during culture on the mechanical properties of the scaffolds. At predefined time points, the samples were retrieved from the culture medium and subjected to immediate mechanical testing. Cell-free scaffolds incubated under identical culture conditions served as controls.

### 2.4. Statistical Analysis

All data are presented as mean ± standard deviation (SD) from three independent experiments, each conducted at least triplicate. Statistical analyses were performed using GraphPad InStat software version 3.10 (GraphPad Software, San Diego, CA, USA). Differences between groups were assessed using two-way analysis of variance (ANOVA) followed by Tukey’s post hoc test. A *p*-value of <0.05 was considered statistically significant.

## 3. Results and Discussion

Several studies have demonstrated that scaffold thickness plays a critical role in tenogenic differentiation [30]. Optimal matrix thickness can be precisely controlled by tuning electrospinning parameters, including spinning duration, flow rate, and nozzle-to-collector distance. In the present study, bilayer PBAT matrices with systematically varied thicknesses and mechanical properties were fabricated as a model for the posterior region of a prospective SSP tendon patch, and their effects on tenogenic differentiation were systematically evaluated. The novelty of this work resides in the application of a bilayer scaffold architecture not to address the tendon–bone interface, but rather to recapitulate the intratendinous structural and mechanical requirements specific to the posterior region of the SSP tendon.

### 3.1. Fabrication and Characterization of Samples

The aligned fiber layer was designed to direct cellular orientation along the primary loading axis, whereas the random fiber layer provided increased porosity to promote diffusion- and capillary-driven transport of nutrients, oxygen, and soluble factors. The bilayer scaffold was fabricated by integrating aligned and randomly oriented fiber layers to achieve functional differentiation rather than thickness equivalence. Interlayer adhesion was established through solvent-mediated interfacial fusion, which enabled polymer chain interdiffusion and physical entanglement between sequentially deposited layers [31].

**Figure 2:**
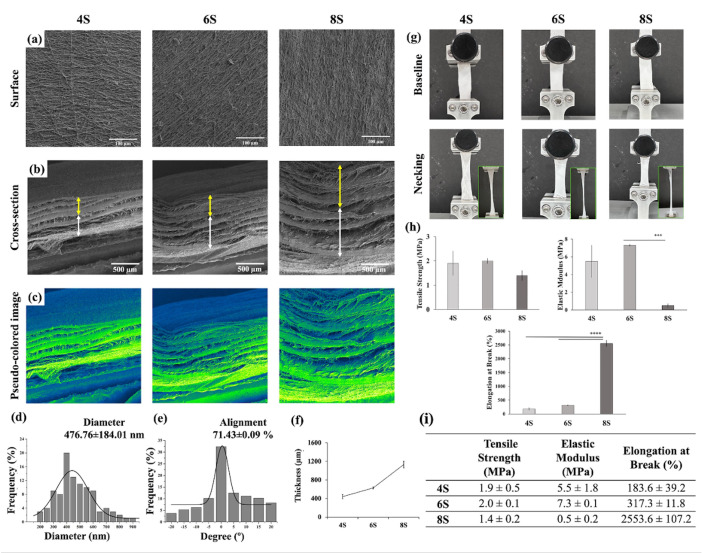
Characterization of the fabricated bilayer scaffolds. (**a**) Surface, (**b**) cross-section, and (**c**) pseudo-colored SEM images of the 4S, 6S, and 8S samples, including higher-magnification micrographs of both scaffold surfaces. In the pseudo-colored images, intensely green regions represent the randomly oriented fiber (basal) layer, which functions as a mechanically supportive structure, whereas lighter green regions correspond to the aligned fiber layer. White arrows indicate the random fiber layers, and yellow arrows denote the aligned fiber layers. (**e**) Fiber diameter distribution, (**d**) fiber alignment distribution (normalized to 90°), and (**f**) scaffold thickness. (**g**) Representative photographic images of the 4S, 6S, and 8S scaffolds during tensile testing, with the first row showing the undeformed state and the second row illustrating necking and elongation behavior. (**h**) Tensile strength, elastic modulus, and elongation at break of the samples. Statistically significant differences among groups are indicated (*n* = 3; ^✤✤✤^ *p* < 0.001 and ^✤✤✤✤^ *p* < 0.0001 among groups. (**i**) Summary table of the measured mechanical properties.

Aligned fibers (Figure 2a) and cross-sections (Figure 2b) of the bilayer PBAT matrices were visualized using SEM. Pseudo-colored images were generated from the original SEM micrographs using ImageJ software to enhance visual contrast (Figure 2c). The distribution of aligned fibers is presented in Figure 2d, with an average diameter of 476.8 ± 184.0 nm. Random fibers exhibited an average diameter of 740.3 ± 180.2 nm. Fiber alignment was normalized to a 90° reference axis. As shown in Figure 2e, 71.4 ± 0.1% of the aligned nanofibers were oriented within the −20° to +20° range. Both fiber diameter and alignment were quantified using ImageJ software. Variations in scaffold thickness corresponding to different electrospinning durations are illustrated in Figure 2f. The measured total thicknesses of the 4S, 6S, and 8S samples were 441.1 ± 45.3 µm (aligned layer: 110.3 ± 11.7 µm and random layer: 330.8 ± 17.1 µm), 629.0 ± 22.0 µm (aligned layer: 157.3 ± 5.7 µm and random layer: 471.8 ± 17.1 µm), and 1132.2 ± 71.2 µm (aligned layer: 283.1 ± 18.4 µm and random layer: 849.2 ± 55.2 µm), respectively.

Tendons act as “mechanical bridges” transmitting forces from muscles to bones; thus, scaffolds for tendon tissue engineering must exhibit suitable mechanical properties. An ideal tendon scaffold should possess high tensile strength and flexibility under load [32]. The mechanical performance of scaffolds with varying thicknesses is shown in Figure 2h,i. The tensile strength values for the 4S, 6S, and 8S samples were 1.9 ± 0.5 MPa, 2.0 ± 0.1 MPa, and 1.4 ± 0.2 MPa, respectively. While the 4S and 6S samples exhibited similar tensile strengths, the substantial reduction in the 8S sample suggests a thickness-dependent decrease occurring beyond a certain threshold. Comparable trends have been reported by Taele et al. [33] and Cao et al. [34], who observed higher flexural strength in thinner carbon fiber laminates.

The elastic moduli of scaffolds were 5.5 ± 1.8 MPa (4S), 7.3 ± 0.1 MPa (6S), and 0.5 ± 0.2 MPa (8S), indicating that the 4S and 6S samples had comparable stiffness, with a slight increase in 6S, whereas the modulus dropped sharply in the thickest sample (8S). The highest elongation at break was observed in the 8S group (2553.6 ± 107.2%), followed by 6S (317.3 ± 11.8%) and 4S (183.6 ± 39.2%). Overall, elongation increased substantially with thickness. Although this trend generally corresponded to a decrease in elastic modulus, the relationship was not strictly inverse, as the 6S samples, despite having a higher modulus than 4S, exhibited greater elongation.

### 3.2. Biodegradation of the Samples

**Figure 3:**
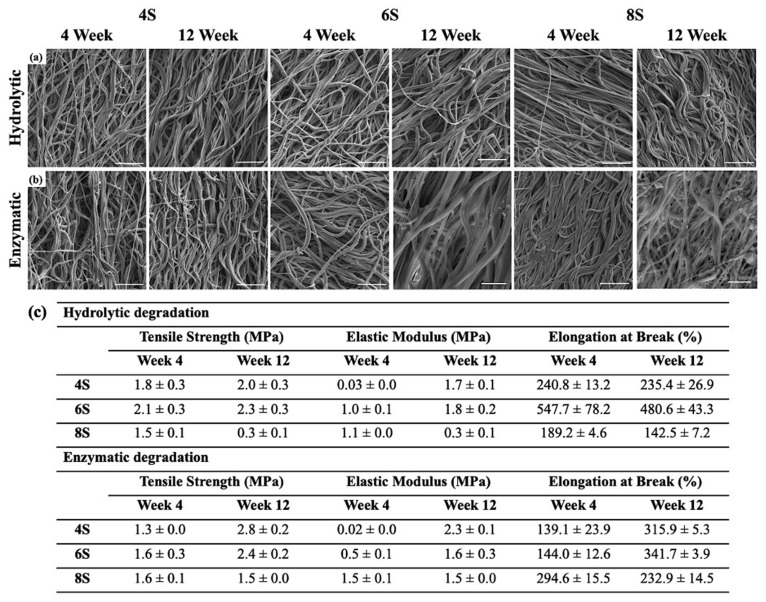
SEM images of the 4S, 6S, and 8S samples after (**a**) hydrolytic and (**b**) enzymatic degradation at the 4th and 12th weeks (scale bars = 10 µm). (**c**) Mechanical properties of the bilayer matrices after hydrolytic and enzymatic degradation at the 4th and 12th weeks.

The biodegradation behavior of PBAT bilayer nanofiber scaffolds was strongly influenced by scaffold thickness and environmental conditions. In agreement with previous findings [35,36], PBAT underwent ester bond cleavage through hydrolytic ion attack and lipase-mediated enzymatic activity, resulting in progressive surface erosion. PBAT does not exhibit the rapid degradation seen in polymers such as PLA or PGA. Its slower, controlled degradation limits terephthalic acid (TPA) release to levels within tissue clearance capacity, reducing local accumulation and toxicity risk. Degradation yields small amounts of low-molecular-weight byproducts, mainly adipic acid and TPA, which show low to moderate toxicity and are systemically cleared. Consequently, they do not induce a significant inflammatory response under physiologically relevant conditions [37,38]. As shown in Figure 3a,b, progressive degradation led to pronounced fiber entanglement and localized fusion, particularly on the aligned surface, resulting in compact bundle formation, a phenomenon consistent with previously reported PBAT surface-remodeling behavior [39,40]. The graphical form of the tensile properties after biodegradation was given in Supplementary Figure S1.

Under hydrolytic conditions, distinct thickness-dependent mechanical responses emerged (Figure 3c). The statistical significance values associated with these mechanical changes are reported comprehensively in Table S2. Across groups, 4S retained its initial tensile strength (1.9 ± 0.5 MPa) until week 12 (2.0 ± 0.3 MPa), despite a transient modulus decrease at week 4; this decrease subsequently recovered, indicating that structural remodeling had occurred following a transient softening. 6S matrices retained comparable strength (initial 2.0 ± 0.1 MPa; week 12: 2.3 ± 0.3 MPa) and showed a significant increase in elongation (from 317.3% to ~480%; * *p* < 0.05), thereby achieving a more ductile, tendon-like profile. In contrast, thick 8S matrices showed statistically significant decreases in tensile strength and modulus by week 12 (e.g., strength from 1.4 ± 0.2 MPa to 0.3 ± 0.1 MPa; modulus from 0.5 ± 0.2 MPa to 0.3 ± 0.1 MPa; * *p* < 0.05), depending upon surface erosion of PBAT nanofibers.

Enzymatic degradation caused an initial softening in all groups due to surface bond rupture, followed by thickness-dependent pattern (Figure 3c). The tensile strength, elongation and elastic modulus values of 4S matrices increased significantly between the 4th and 12th weeks (* *p* < 0.05) and ultimately exceeded the initial values (strength increased from 1.3 MPa to 2.8 MPa; elastic modulus increased from 0.02 MPa to 2.3 MPa). This can be explained by the selective separation of weak fiber segments from the matrix by effective enzyme infiltration, resulting in a mechanically strengthened matrix. Although significant increases in strength and elongation were also seen in 6S matrices (* *p* < 0.05), there was a lesser mechanical improvement in the structure than that of 4S (strength increased from 1.6 to 2.4 MPa). Meanwhile, 8S showed no significant change in strength and elastic modulus values and only a significant decrease in elongation (* *p* < 0.05); This can be explained by limited enzyme penetration and a predominantly superficial change.

Overall, 4S allowed controlled adaptation to maintain mechanical integrity in both hydrolytic and enzymatic environments. The observed increase in mechanical properties is attributed to structural reorganization rather than intrinsic material strengthening. During incubation, interactions between the liquid and polymer chains induced surface plasticization, promoting fiber entanglement and interlocking. This effect led to increased tensile strength, particularly in thinner scaffolds. 6S matrices exhibited stable hydrolytic behavior and moderate enzymatic response, while 8S was the most susceptible to degradation. Based on these results, 4S was identified as the most promising configuration for the SSP tendon matrix.

### 3.3. Cell Culture Results

#### 3.3.1. Determination of Cell Viability

Tendons are characterized by inherently low cellularity, which significantly limits their intrinsic regenerative capacity. Although tenocytes and fibroblasts are commonly used in tendon tissue engineering, isolating primary tenocytes directly from tendon tissue is technically challenging and often yields limited cell numbers [41,42]. In the present study, rat adipose-derived mesenchymal stem cells (r-AdMSCs) were selected as the cellular source. Flow cytometric analysis confirmed the mesenchymal phenotype of the cells, showing positive expression of CD29, CD90, CD106, and CD54, and negative expression of CD45 and CD11b, consistent with Dominici’s criteria. The cells exhibited a doubling time of 44 h (specific growth rate: 0.0157 h^−1^) and successfully differentiated into adipogenic, chondrogenic, and osteogenic lineages under appropriate induction conditions [26]. These cells are widely preferred in tendon tissue engineering due to their ease of isolation, high proliferative potential, and strong tenogenic differentiation capacity [30,43].

**Figure 4:**
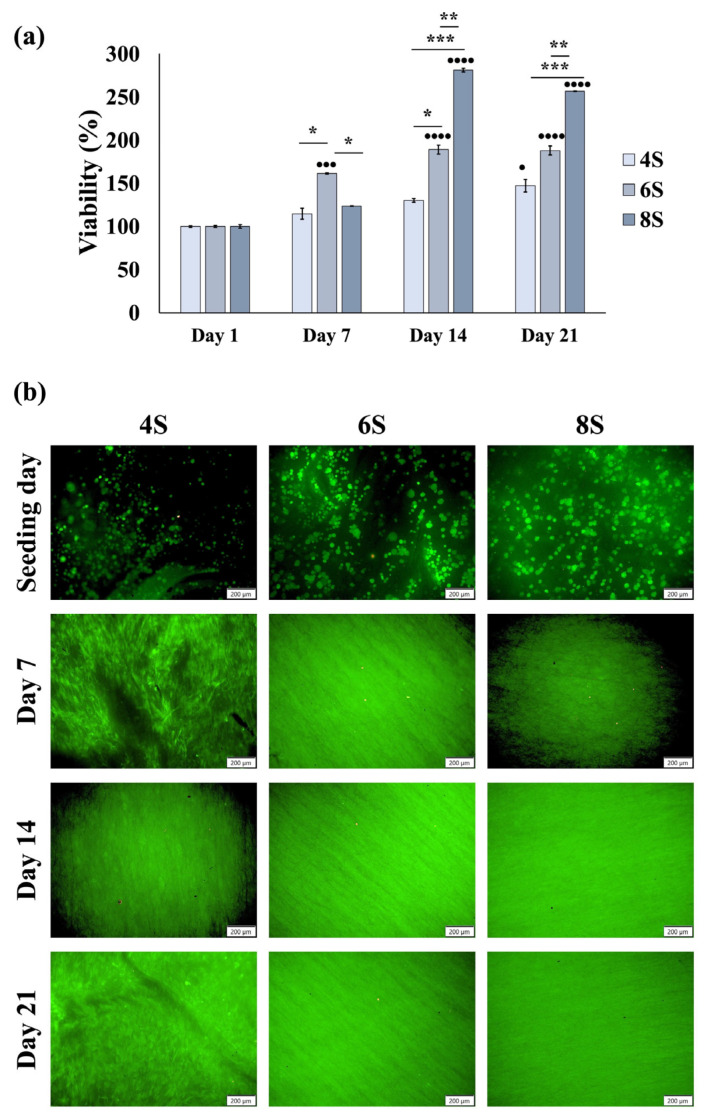
Viability of cells on scaffolds with different thicknesses and their effect on scaffold mechanical properties (**a**) MTT analysis result of r-AdMSCs on scaffolds at different time intervals (Statistically significant differences, *n* = 3, ^•^ *p* < 0.05, ^•••^ *p* < 0.001, and ^••••^ *p* < 0.0001 when day 1 was taken as the control and normalized to 100% viability for each group; * *p* < 0.05, ** *p* < 0.01 and *** *p* < 0.001 when the groups compared on same day), (**b**) live/dead staining fluorescent images of r-AdMSCs on samples at different time intervals. Calcein AM shows live cells as green and ethidium homodimer demonstrates cells as red. Scale bars represent 200 µm.

Within this biological context, r-AdMSCs proliferation on 4S, 6S, and 8S PBAT bilayer scaffolds demonstrated distinct thickness-dependent patterns over the 21-day culture period (Figure 4a). Normalizing each group’s first-day value as internal control enabled a more precise interpretation of how scaffold thickness modulates cellular behavior.

The 4S scaffolds showed a steady increase in cell proliferation throughout the culture period. While the increase in the early phase was not statistically significant, a significant increase was observed over the following days, reaching significance on day 21 (^•^ *p* < 0.05). This gradual and moderate proliferation is consistent with the physiological characteristics of promotion of tenogenic phenotype, where tenocyte-like cells are expected to exhibit orderly growth that maintains their phenotype rather than rapid, uncontrolled proliferation. Thus, the 4S scaffold appears to provide a favorable microenvironment supporting early cellular alignment, morphological organization, and maintenance of a tenocyte-like phenotype.

In contrast, 6S scaffolds exhibited more pronounced proliferation at the early culture stage, with statistically significant increases observed at all time points (^•••^ *p* < 0.001) at the early stage; ^••••^ *p* < 0.0001 at the middle and late stages). While rapid proliferation is desirable for ECM depositions, it is known that excessively rapid proliferation can delay tenogenic differentiation in some cases. However, because the 6S scaffold exhibited relatively stable proliferation at later stages, it is still considered a suitable candidate for tenogenic tissue engineering.

8S scaffolds exhibited limited proliferation in the early phase, followed by a sharp and statistically significant increase in the mid- and late phases (^••••^ *p* < 0.0001). However, this late-stage proliferative increase may indicate an undesirable hyperproliferative response in the context of tendon tissue engineering. Excessive proliferation is associated with a shift toward a fibroblast-like phenotype, impaired collagen organization, and slower tenogenic maturation.

Live/dead staining was performed to confirm the MTT results. Figure 4b shows fluorescence images from the day of transplantation and days 7, 14, and 21. It is seen that there were many live cells in all groups, and the cells proliferated along the direction of the aligned nanofibers, consistent with tendon tissue engineering studies in the literature [30,41].

These findings demonstrate that scaffold thickness is a key determinant of r-AdMSCs behavior, influencing not only proliferation dynamics but also the tenogenic commitment. Among the tested constructs, the 4S bilayer scaffold promotes the most physiologically aligned, phenotype-preserving proliferation profile, making it a compelling candidate for tendon tissue engineering applications.

It is well established that mesenchymal stem cells may transiently reduce proliferation, often due to cell cycle arrest, prior to lineage commitment [44]. Accordingly, the lower proliferation observed in 4S scaffolds may reflect a shift in cellular activity from division toward tenogenic differentiation, including expression of markers such as Tnmd and Scx, and ECM organization. The 4S architecture, with optimized thickness and fiber density, likely provided more effective mechanical cues, promoting mechanotransduction associated with tenogenic commitment. In contrast, the 8S group may have introduced excessive cell density or less favorable matrix properties, limiting such cues and favoring maintenance or non-specific proliferation rather than differentiation. Thus, r-AdMSCs in 4S scaffolds likely redirected metabolic resources toward adhesion, migration along aligned fibers, and activation of lineage-specific gene expression, as supported by the higher tenogenic marker expression compared to 8S.

#### 3.3.2. RT-qPCR Analysis

**Figure 5:**
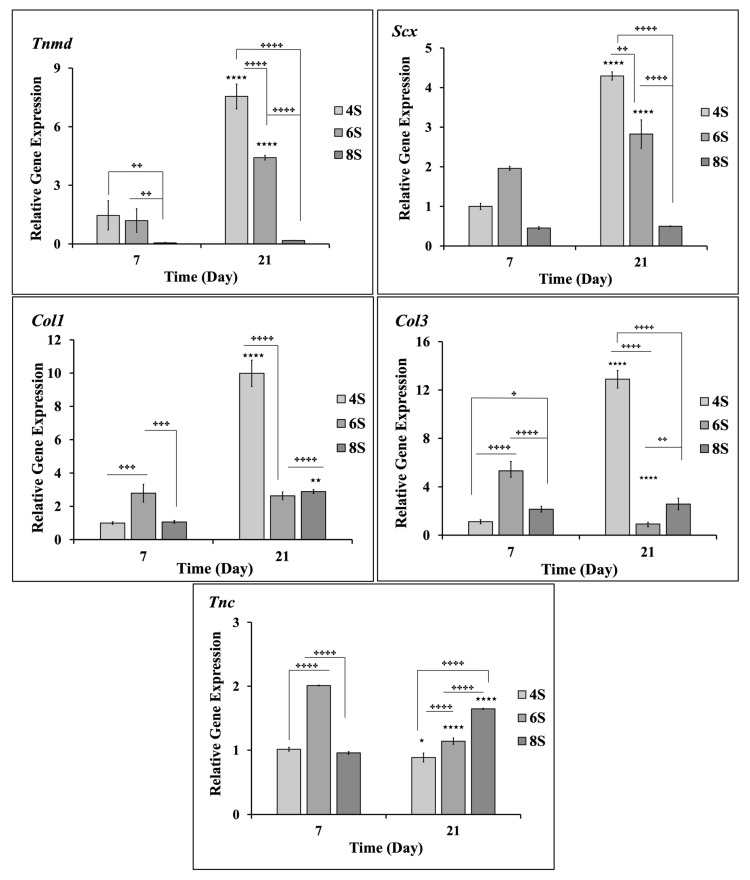
RT-qPCR analysis results of r-AdMSCs at different time points (Statistically significant differences, *n* = 4, ^✤^ *p* < 0.05, ^✤✤^ *p* < 0.01, ^✤✤✤^ *p* < 0.001, and ^✤✤✤✤^ *p* < 0.0001 among the groups on the same day; ^★^ *p* < 0.05, ^★★^ *p* < 0.01 and ^★★★★^ *p* < 0.0001 within the same group on the following days).

The effect of matrix thickness on the tenogenic commitment of r-AdMSCs under in vitro conditions was evaluated by RT-qPCR analysis of tendon specific markers (Tnmd, Tnc, Scx, Col1, and Col3), and the results are represented in Figure 5.

Tenomodulin (Tnmd), a type II transmembrane glycoprotein expressed in dense connective tissues such as tendons and ligaments, is localized along aligned type I collagen fibers, the predominant ECM component of tendons. These fibers organize elongated tenocytes into parallel layers, while oval tenocytes are found in the interlaminar spaces. Because Tnmd expression is specific to elongated tenocytes, it is considered a reliable marker of mature tendon tissue [43]. As seen in Figure 5, Tnmd expression on day 7 was significantly higher in the 4S and 6S groups compared to the 8S group (^✤✤^ *p* < 0.01). On day 21, the 4S group showed the highest Tnmd levels (^✤✤✤✤^ *p* < 0.0001 and ^★★★★^ *p* < 0.0001), with approximately 8-fold increase compared to 6S. Given the role of Tnmd in tendon-like reparative tissue formation and collagen fiber organization, these results indicate significantly increased tenogenic maturation in the 4S scaffold.

Scx, another transcription factor indicative of new tendon tissue formation, is known to respond to mechanical loading [45]. Although no significant difference was seen in initial Scx expression in 4S, its level increased approximately 4-fold by day 21 (^★★★★^ *p* < 0.0001), mirroring the trend seen in Tnmd. The 6S group also exhibited significant upregulation at day 21 (^★★★★^ *p* < 0.0001), although 4S remained statistically approximately 1-fold higher (^✤✤^ *p* < 0.01). Co-expression of Scx and Tnmd is critical for tendon development and maintenance, and both genes are upregulated in injured tendons [46]. Numerous studies confirm simultaneous Scx-Tnmd expression during tendon development [47,48,49,50,51,52]. In this study, significantly higher Scx and Tnmd levels in the 4S group coincided with improved tensile strength and elastic modulus values.

In this study, mechanical cues were evaluated within the framework of the passive mechanical microenvironment, including scaffold elastic modulus, thickness, and fiber alignment. Although specific intracellular mechanotransduction pathways—such as Yes-associated protein (YAP)/transcriptional co-activator with PDZ-binding motif (TAZ) signaling or integrin-mediated pathways—were not directly examined, the thickness-dependent modulation of elastic modulus, together with the concomitant upregulation of *Scx* and *Tnmd*, provides functional evidence of mechanically driven tenogenic differentiation [49,50,51,52,53].

Col1 and Col3, the key structural components of the tendon ECM, also show thickness-dependent expression [48]. Figure 5 shows that Col1 and Col3 followed similar trends in all groups. For Col1, the 4S group showed significantly lower expression than 6S on days 7 and 21 (^✤✤✤^ *p* < 0.001 and ^✤✤✤✤^ *p* < 0.0001), but expression increased almost 10-fold on day 21 (^★★★★^ *p* < 0.0001). No significant temporal change occurred in 6S (^★^ *p* < 0.05), while 8S showed only a 1-fold increase on day 21 (^★★^ *p* < 0.01). A comparable trend was observed for Col3 as well as Col1. 6S had the highest early expression at day 7 (^✤✤✤✤^ *p* < 0.0001), but expression decreased 4-fold (^★★★★^ *p* < 0.0001) by day 21. Conversely, the 4S group showed the highest Col3 expression at day 21, with a 12-fold increase compared to the other scaffolds (^✤✤✤✤^ *p* < 0.0001). Our findings regarding Tnmd, Scx, Col1 and Col3 expression are consistent with similar study results [42,48] and support that the 4S scaffold is a promising candidate for supraspinatus tendon patch applications.

Tnc, another key tendon marker, exhibits dual expression patterns: one associated with the organized fibrous matrix of healthy tendon, contributing to collagen fibril organization, and another linked to degenerated tendons where Tnc is absent in poorly organized fibrocartilaginous ECM [54]. Although its role in tendon degeneration remains unclear, Tnc is involved in ECM organization and cell proliferation, and its expression increases following mechanical overload [55]. As shown in Figure 5, Tnc levels in the 4S group were ~1-fold lower than in 6S on day 7 (^✤✤✤✤^ *p* < 0.0001). The 6S group exhibited significantly elevated Tnc expression at day 7 that declined by day 21 (^★★★★^ *p* < 0.0001), potentially indicating the end of the remodeling stage. In contrast, the thickest scaffold, 8S, showed the highest Tnc expression at day 21 (^✤✤✤✤^ *p* < 0.0001 and ^★★★★^ *p* < 0.0001). However, this Tnc upregulation must be interpreted alongside the very low Col and Tnmd expression observed in 8S. High Tnc combined with low tenogenic markers suggests remodeling or degeneration rather than healthy tendon formation. Although Tnc upregulation in degeneration or remodeling is recognized, its precise regenerative role remains unclear. Chen et al. reported that GDF-5–induced adipose-derived MSC sheets exhibited higher Tnc expression in nanoyarn scaffolds [56], consistent with GDF-5’s role in tendon repair [57]. In the current study, promotion of tenogenic phenotype was evaluated without exogenous growth factors or differentiation cues, focusing solely on scaffold thickness. The highest expression of genes critical for tendon growth and repair, together with superior tensile strength and elasticity, was observed in the 4S samples.

#### 3.3.3. Immunofluorescence Analysis of Cell Morphology and Tenogenic Markers

**Figure 6:**
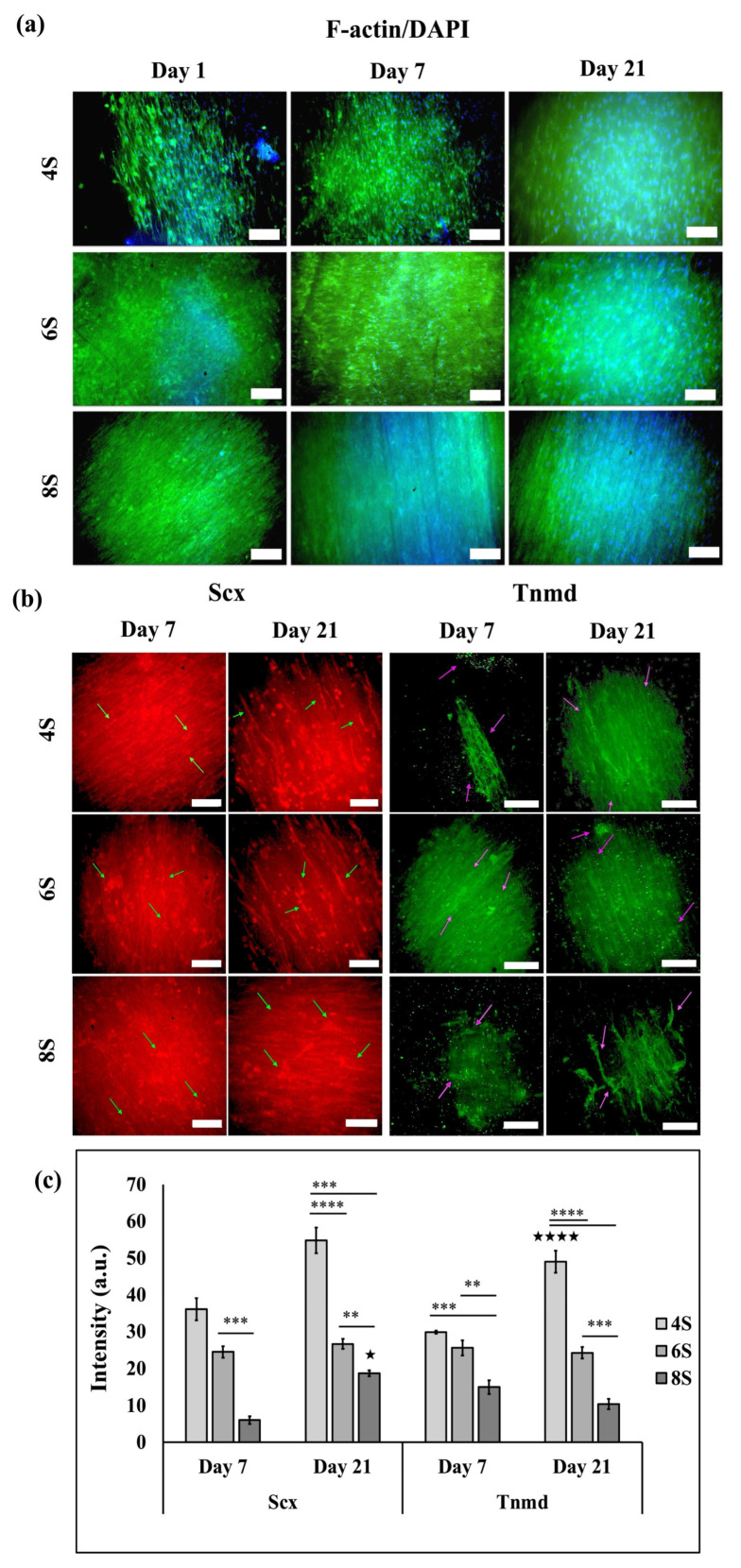
Fluorescence microscopy images of rat adipose-derived mesenchymal stem cells (r-AdMSCs) cultured on scaffolds at predetermined time points. (**a**) F-actin staining (green) illustrating the cytoskeletal organization and DAPI staining (blue) indicating cell nuclei (scale bars: 200 µm for day 1 and day 7; 100 µm for day 21). (**b**) Scx–Alexa Fluor^®^ 594 immunostaining, with green arrows indicating Scx-expressing cells on the matrices. Tnmd–Alexa Fluor^®^ 488 immunostaining, with purple arrows highlighting ECM-associated labeling (scale bars for Scx and Tnmd: 100 µm). (**c**) Quantification of fluorescence intensity for Scx and Tnmd in 4S, 6S, and 8S scaffolds at day 7 and day 21. Statistical significance is indicated as ** *p* < 0.01, *** *p* < 0.001, and **** *p* < 0.0001 for intergroups comparisons and ^★^ *p* < 0.05 and ^★★★★^ *p* < 0.0001 for intragroup comparisons.

The cytoskeleton and nuclei of r-AdMSCs were monitored by F-actin/DAPI staining at predetermined days of culture, and the images are shown in Figure 6a. On day 1, cells migrated along the fiber direction and attached to the surface in all samples. Due to their increased thickness in the 6S and 8S samples, the cells began to spread inward. However, in the 4S group, the actin filaments of the cells were more aligned along the fiber direction compared to the 6S and 8S samples. This may be attributed to the decrease in the gaps between the fibers with increasing sample thickness. On day 7, the cells maintained their alignment and proliferated on the surface. Furthermore, the elliptical nuclei of the cells were considered a clue of tenogenic phenotype [57].

The antibodies, Scx and Tnmd are tendon specific markers and are considered an indicator for tenogenic differentiation [57]. Anti-Scx immunohistochemistry staining was performed on the 7th and 21st days of the culture and the images were presented in Figure 6b. Scx is localized in cell nuclei at early stages and found in cell cytoplasm at later stages after commitment [58]. Figure 6b displays the staining of Scx at the cell nuclei, indicated by green arrows on the 7th day for all groups. The RT-qPCR results supported the higher staining intensity observed in the 4S and 6S samples compared to the 8S samples. On the 21st day, Scx was detected in the cell cytoplasm and visualized intensely in the 4S and 6S samples. The alignment of cells was apparent through fibers. Since Scx relates to the mechanical response of the tendon, it can express itself more in dynamical reactor system conditions.

Anti-Tnmd immunostaining was performed on days 7 and 21, and representative images are presented in Figure 6c. Consistent with the RT-qPCR results shown in Figure 5, cells cultured on the 4S scaffolds exhibited more intense Tnmd staining compared with those on the 6S and 8S scaffolds. In native tendon, Tnmd is localized in the ECM and aligns along collagen fibers. In this study, Tnmd was visualized using AlexaFluor^®^ 488 (green), co-aligned with collagen bundles indicated by purple arrows. On day 7, the 6S group showed weaker staining than the 4S group, which agreed with the corresponding RT-qPCR data. Additionally, RT-qPCR analysis revealed that the 8S group secreted almost no Tnmd at this time point. By day 21, elongated Tnmd-positive structures became more prominent in both the 4S and 6S groups, further supporting the gene expression findings. As seen on Figure 6c, as expected, 4S exhibited the most intense fluorescence for both antibodies than the 6S and 8S (*** *p* < 0.001 and **** *p* < 0.0001 for Scx and **** *p* < 0.0001 at day 21). This result correlated with the RT-qPCR analysis.

#### 3.3.4. Results of Post-Culture Tensile Tests

Mechanical tests (Table 1, Supplementary Figure S2, Supplementary Table S3) showed that the scaffolds underwent significant restructuring in culture, even in the absence of cells. This is thought to be due to passive hardening due to medium proteins, ions, and hydration, particularly in the 6S and 8S groups, as well as fiber compression due to adsorption, hydrolysis, and swelling. This suggests that PBAT networks, especially in thicker structures, are highly sensitive to physicochemical cues and can undergo structural compression without any cell-based biological contribution.

Under cell-free conditions, only 6S exhibited a significant increase in tensile strength over time (**** *p* < 0.0001), despite an initial transient decrease (* *p* < 0.05). This early decline is likely due to hydration-induced fiber loosening, where water absorption weakens fiber–fiber interactions and causes slight separation, resulting in a brief period of mechanical instability. In cell-laden scaffolds, both 4S and 6S showed progressive strengthening over time (* *p* < 0.05, ** *p* < 0.01). Notably, 4S initially maintained mechanical stability and subsequently increased in strength as cells deposited ECM, suggesting that thinner scaffolds are more influenced by cellular activity rather than ambient conditions for their mechanical performance. The lower tensile strength of 6S compared with 4S and 8S on day 7 (* *p* < 0.01) indicates that medium-thickness scaffolds are more susceptible to early degradation, resulting in reduced mechanical integrity at this stage. By day 21, 8S emerged as the most mechanically resilient scaffold (*p* < 0.05), likely due to the development of swelling-induced passive tension in thicker constructs.

Elongation at break offered deeper mechanistic insight into tendon-like function. In the absence of cells, ductility decreased sharply in 6S and 8S (**** *p* < 0.0001), demonstrating that degradation alone introduces microstructural brittleness. In contrast, cell-laden 4S exhibited significantly increased elongation (** *p* < 0.01; * *p* < 0.05), consistent with imaging and gene expression data showing aligned, collagen rich ECM deposition. This behavior suggests an efficient fiber/ECM load sharing mechanism in 4S, enabling energy dissipating, tendon-like extensibility that neither 6S nor 8S could recreate to the same degree. The fact that 4S maintains high extensibility across conditions indicates that its moderate thickness and aligned microarchitecture create a highly favorable mechanotransductive environment.

**Table 1:** Mechanical properties of PBAT bilayer scaffolds in culture media without cells (cell-free) and with cells (cell-laden).

**Cell-Free**						
	**Tensile Strength (MPa)**		**Elongation at Break (%)**		**Elastic Modulus (MPa)**	
	**Day 7**	**Day 21**	**Day 7**	**Day 21**	**Day 7**	**Day 21**
**4S**	1.6 ± 0.4	1.2 ± 0.1	109.4 ± 7.9	169.7 ± 30.5	5.7 ± 2.2	10.2 ± 0.8
**6S**	0.3 ± 0.1	2.1 ± 0.2	77.4 ± 6.3	129.4 ± 18.0	7.0 ± 2.2	9.3 ± 0.7
**8S**	1.2 ± 0.3	4.4 ± 0.9	89.5 ± 3.8	238.2 ± 48.3	10.7 ± 1.3	5.4 ± 1.4
**Cell-laden**						
	**Tensile Strength (MPa)**		**Elongation at Break (%)**		**Elastic Modulus (MPa)**	
	**Day 7**	**Day 21**	**Day 7**	**Day 21**	**Day 7**	**Day 21**
**4S**	1.0 ± 0.1	2.1 ± 1.4	160.8 ± 18.3	236.8 ± 5.5	6.6 ± 1.0	3.4 ± 0.4
**6S**	0.3 ± 0.1	1.9 ± 1.2	170.4 ± 15.2	122.9 ± 15.7	1.3 ± 0.2	2.2 ± 0.4
**8S**	1.2 ± 0.1	3.6 ± 1.7	191.5 ± 13.9	447.3 ± 50.7	9.9 ± 2.2	2.8 ± 0.2

Elastic modulus changes reinforced the same thickness-dependent trends. In cell-free samples, only 8S stiffened significantly at day 21 (* *p* < 0.05), consistent with constrained swelling and densification typical of thicker fibrous constructs. Under cell-laden conditions, however, the modulus hierarchy reversed: 4S became the stiffest by day 21 (**** *p* < 0.0001), suggesting that r-AdMSCs can exert more effective traction forces and reorganize fibers more coherently in thinner scaffolds. Although 8S also stiffened in the presence of cells (**** *p* < 0.0001), its ECM remained less directionally organized, demonstrating that stiffness alone is not a reliable indicator of tendon-like quality unless supported by oriented collagen architecture.

Under cell-free conditions, PBAT scaffolds passively stiffen and become less ductile due to adsorption, hydrolysis, and swelling; these effects are strongest in 6S and 8S. When cells are present, remodeling becomes active: r-AdMSCs generate force, align fibers, change stiffness, and deposit collagen. These effects are most organized and tendon-like in 4S. The moderately thick 6S remains strongly affected by moderate impacts, limiting cellular remodeling; while 8S undergoes pronounced swelling and volume-driven changes that lead to less aligned ECM despite improved mechanical strength.

In the early stages, ECM components were synthesized by the cells along the fibers. These components acted as a biological binder. The fibers interlocked, forming a composite structure together with the ECM, which filled the gaps between them. This structure may have contributed to and stabilized the mechanical performance prior to degradation at later stages.

These mechanistic pathways align with known SSP requirements moderate tensile strength, high extensibility, and controlled stiffness [3]. Among the tested constructs, 4S most closely recapitulates this profile, whereas 6S changes in response to cellular remodeling and 8S, despite becoming mechanically strong, lacks sufficient ECM directionality for tendon fidelity. The absence of biophysical stimulation represents a key limitation, as mechanical loading may further enhance or clarify these remodeling pathways [59].

## 4. Conclusions

This study demonstrates that bilayer anisotropic PBAT nanofibrous scaffolds with thickness-controlled architectures can effectively direct r-AdMSCs toward a tenogenic phenotype via gene expression, cellular morphology, and mechanical properties. The thicker 8S scaffold exhibited delayed remodeling and weaker mechanical maturation, whereas the intermediate 6S scaffold showed only moderate performance. Among all configurations, the 4S (4 h deposition of aligned layer onto 4 h deposition of random layer; 440 µm thickness, ~70% alignment and ~450 nm fiber diameter) scaffold provided the most favorable balance of mechanical stability, controlled degradation, and biologically instructive cues. This scaffold promoted aligned ECM deposition, enhanced collagen organization, and significantly upregulated tendon-specific markers (Tnmd, Scx, Col1, Col3), collectively indicating superior tenogenic maturation.

Overall, these results underscore scaffold thickness as a key determinant of mechanobiological function, with the 4S configuration emerging as the most promising candidate for tendon-mimetic applications. Taken together, the findings position PBAT-based anisotropic bilayer scaffolds-particularly the 4S design- as strong candidates for further evaluation under dynamic bioreactor conditions and in vivo models. Future studies incorporating bioreactor-mediated biophysical stimulation and/or biochemical stimulation with GDF-5, followed by in vivo validation, will further enhance their translational potential.

## Data Availability

The data presented in this study are available from the corresponding author upon reasonable request.

## Supplementary Materials

The following supporting information can be downloaded at: https://www.mdpi.com/article/10.3390/jfb17070310/s1, Table S1: Primers used in RT-qPCR analysis for tenogenic differentiation, Figure S1: Mechanical properties of scaffolds after hydrolytic and enzymatic degradation (a) tensile strength, (b) elastic modulus, and (c) elongation at break (%), Table S2: Statistical differences between hydrolytic and enzymatic degradation samples at weeks 4 and 12, Figure S2: Mechanical properties of scaffolds after cell culture studies (a) tensile strength, (b) elastic modulus, and (c) elongation at break (%), Table S3: Statistical differences between cell-free and cell-laden samples.
