# Supplementary material for: Thickness-Tunable Bilayer PBAT Nanofibrous Scaffolds for Enhancing r-AdMSCs’ Tenogenic Commitment in Supraspinatus Tendon Regeneration
Source: J Funct Biomater. 2026 Jun 23;17(7):310. doi: 10.3390/jfb17070310 (PMC13412227; doi:10.3390/jfb17070310)
Supplement: Supplementary file 1 [file jfb-17-00310-s001.zip › jfb-4333199-supplementary.pdf]

SUPPLEMENTARY MATERIALS

Table S1. Primers used in RT-qPCR analysis for tenogenic differentiation.

**Gen**		**Forward** 5'-3'	**Reverse** 5'-3'	T_a
**Collagen Type I α-1**	*Col1*	CCCAGCGGTGGTTATGACTT	AACGGCCACCATCTTGAGAC	56
**Collagen Type III α-1**	*Col3*	AGTGGCCATAATGGGGAACG	CAGGGTTTCCATCCCTTCCG	57
**Tenomodulin**	*Tnmd*	ATGGGTGGTCCCACAAGTGAA	CTCTCATCCAGCATGGGATCAA	57
**Scleraxis**	*Scx*	AGAACACCCAGCCCAAACAG	TGTCACGGTCTTTGCTCAACT	55
**Tenascin C**	*Tnc*	CGCAAAAATGGACGTGAGGA	AGGTTATCCAGTCCAAGCCAG	55
**β-actin**	*β-actin*	AGCAAGCAGGAGTACGATGAG	AAAGGGTGTAAAACGCAGCTC	56

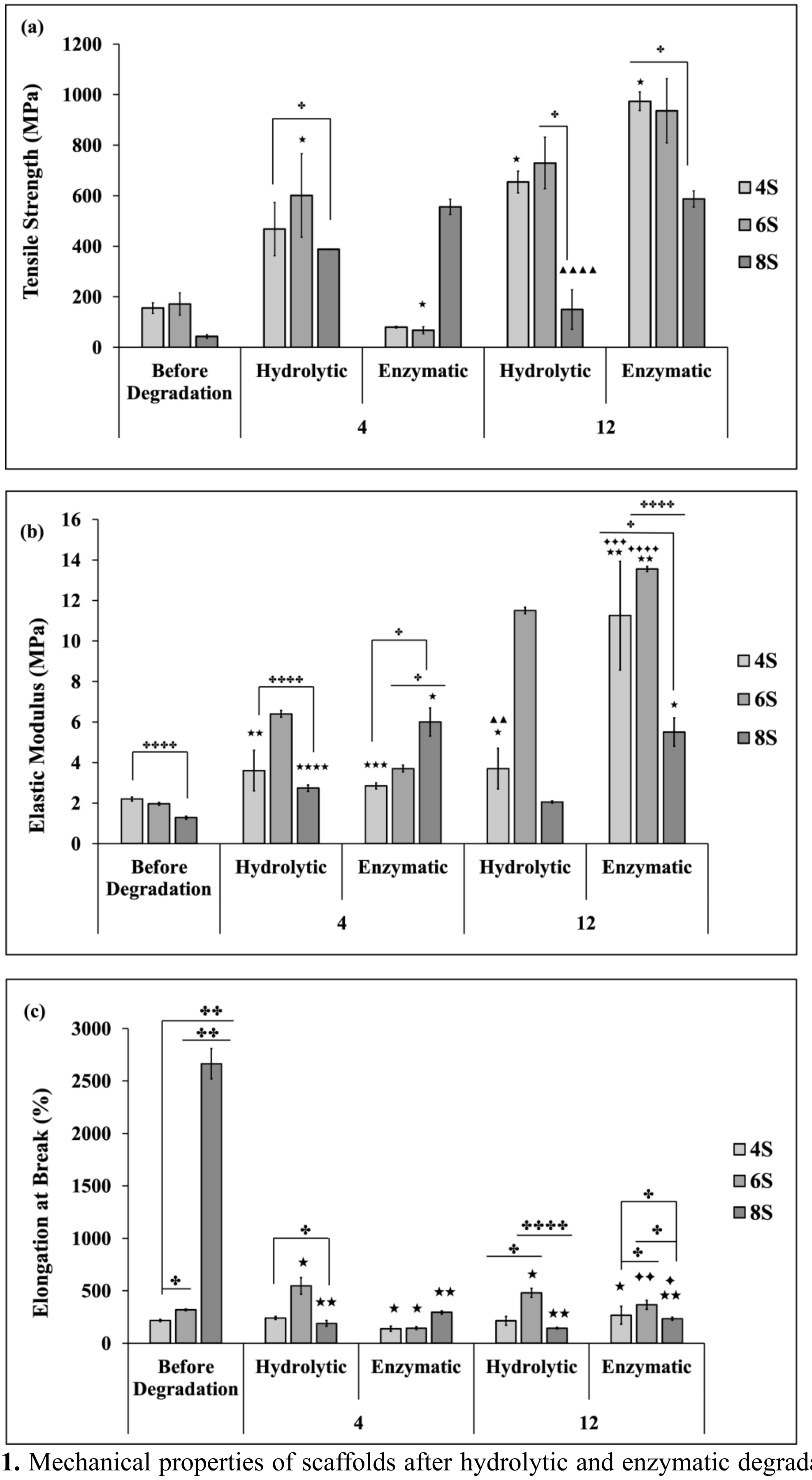

Figure S1. Mechanical properties of scaffolds after hydrolytic and enzymatic degradation (a) tensile strength, (b) elastic modulus, and (c) elongation at break (%)

Table S2. Statistical differences between hydrolytic and enzymatic degradation samples at weeks 4 and 12.

	**Tensile Strength**		**Elastic Modulus**		**Elongation at Break**	
	Statistical difference		Statistical difference		Statistical difference	
**Hydrolytic**	Baseline 8S<br>vs<br>8S (week 12)<br>--<br>4S (week 12)<br>vs<br>8S (week 12)<br>--<br>6S (week 12)<br>vs<br>8S (week 12)	*$p < 0.05$	Baseline 8S<br>vs<br>8S (week 12)<br>--<br>4S (week 12)<br>vs<br>8S (week 12)	*$p < 0.05$	Baseline 8S<br>vs<br>8S (week 4)<br>--<br>Baseline 8S<br>vs<br>8S (week 12)<br>--<br>8S (week 4)<br>vs<br>8S (week 12)<br>--<br>6S (week 12)<br>vs<br>8S (week 12)	*$p < 0.05$
	**Tensile Strength**		**Elastic Modulus**		**Elongation at Break**	
	Statistical difference		Statistical difference		Statistical difference	
**Enzymatic**	4S (week 4)<br>vs<br>4S (week 12)<br>--<br>4S (week 12)<br>vs<br>8S (week 12)<br>--<br>6S (week 12)<br>vs<br>8S (week 12)	*$p < 0.05$	Baseline 4S<br>vs<br>4S (week 4)	**$p < 0.01$	Baseline 6S<br>vs<br>6S (week 4)	**$p < 0.01$
			Baseline 6S<br>vs<br>6S (week 4)<br>--<br>4S (week 4)<br>vs<br>6S (week 4)<br>--<br>4S (week 4)<br>vs<br>8S (week 4)	*$p < 0.05$	Baseline 8S<br>vs<br>8S (week 4)<br>--<br>Baseline 8S<br>vs<br>8S (week 12)<br>--<br>4S (week 4)<br>vs<br>4S (week 12)<br>--<br>8S (week 4)<br>vs<br>8S (week 12)	*$p < 0.05$
			4S (week 4)<br>vs<br>8S (week 12)	***$p < 0.001$		
			6S (week 4)<br>vs<br>8S (week 4)<br>--<br>4S (week 12)<br>vs<br>8S (week 12)	*$p < 0.05$		

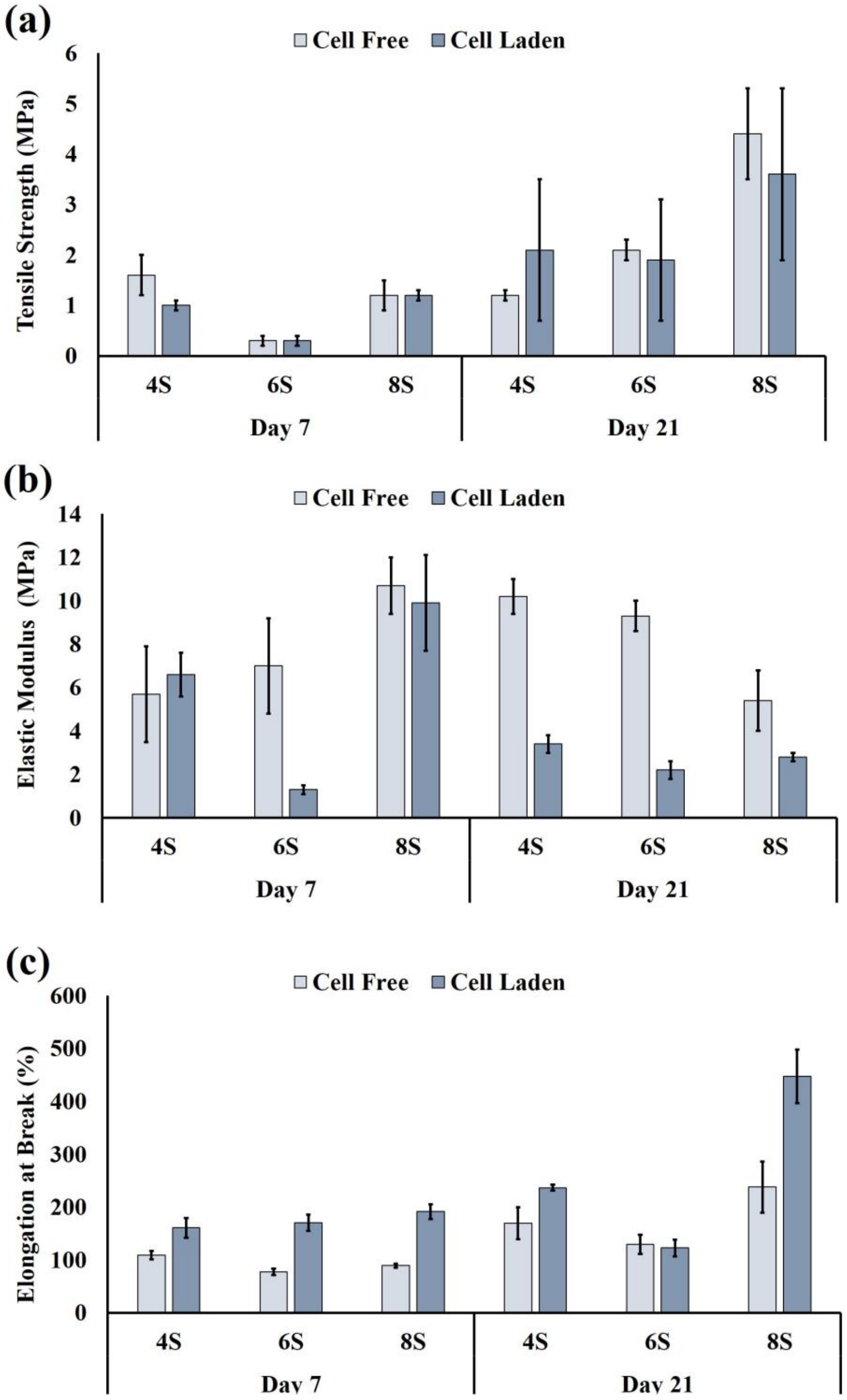

Figure S2. Mechanical properties of scaffolds after cell culture studies (a) tensile strength, (b) elastic modulus, and (c) elongation at break (%).

Table S3. Statistical differences between cell-free and cell-laden samples.

<table>
<tr><td rowspan="5">Cell-free</td><td colspan="2">Tensile Strength</td><td colspan="2">Elastic Modulus</td><td colspan="2">Elongation at Break</td></tr>
<tr><td colspan="2">Statistical difference</td><td colspan="2">Statistical difference</td><td colspan="2">Statistical difference</td></tr>
<tr><td>Baseline 6S
vs
6S (day 7)
--
6S (day 7)
vs
8S (day 7)</td><td>*$p<0.05$</td><td>Baseline 8S
vs
8S (day 7)
--
4S (day 21)
vs
6S (day 21)
--
6S (day 21)
vs
8S (day 21)</td><td>*$p<0.05$</td><td>Baseline 6S
vs
6S (day 7)
--
Baseline 8S
vs
8S (day 7)
--
Baseline 6S
vs
6S (day 21)
--
Baseline 8S
vs
8S (day 21)</td><td>****$p<0.0001$</td></tr>
<tr><td>4S (day 21)
vs
6S (day 21)
--
6S (day 21)
vs
8S (day 21)</td><td>**$p<0.01$</td><td rowspan="2">6S (day 7)
vs
6S (day 21)</td><td rowspan="2">****$p<0.0001$</td><td>4S (day 7)
vs
8S (day 7)
--
6S (day 7)
vs
8S (day 7)</td><td>*$p<0.05$</td></tr>
<tr><td>6S (day 7)
vs
6S (day 21)</td><td>****$p<0.0001$</td><td>4S (day 21)
vs
6S (day 21)
--
6S (day 21)
vs
8S (day 21)</td><td>**$p<0.01$</td></tr>
<tr><td rowspan="3">Cell-laden</td><td colspan="2">Tensile Strength</td><td colspan="2">Elastic Modulus</td><td colspan="2">Elongation at Break</td></tr>
<tr><td colspan="2">Statistical difference</td><td colspan="2">Statistical difference</td><td colspan="2">Statistical difference</td></tr>
<tr><td>Baseline 4S
vs
4S (day 21)
--
Baseline 6S
vs
6S (day 7)
--
Baseline 6S
vs
6S (day 21)
--
4S (day 21)
vs
8S (day 21)
--
6S (day 21)
vs
8S (day 21)
--
4S (day 7)
vs
4S (day 21)</td><td>*$p<0.05$</td><td>6S (day 21)
vs
8S (day 21)
--
4S (day 7)
vs
4S (day 21)
--
6S (day 7)
vs
6S (day 21)</td><td>*$p<0.05$</td><td>Baseline 6S
vs
6S (day 7)
--
Baseline 6S
vs
6S (day 21)
--
4S (day 21)
vs
6S (day 21)
--
6S (day 21)
vs
8S (day 21)</td><td>*$p<0.05$</td></tr>
</table>

	--<br>6S (day 7)<br>vs<br>6S (day 21)					
	4S (day 7)<br>vs<br>6S (day 7)<br>--<br>6S (day 7)<br>vs<br>8S (day 7)	**p<0.01	Baseline 6S<br>vs<br>6S (day 7)<br>--<br>4S (day 21)<br>vs<br>6S (day 21)<br>--<br>Baseline 8S<br>vs<br>8S (day 21)	****p<0.0001	4S (day 7)<br>vs<br>6S (day 7)<br>--<br>6S (day 7)<br>vs<br>8S (day 7)	**p<0.01
			Baseline 6S<br>vs<br>6S (day 21)<br>--<br>4S (day 7)<br>vs<br>6S (day 7)<br>--<br>6S (day 7)<br>vs<br>8S (day 7)	**p<0.01	Baseline 8S<br>vs<br>8S (day 7)<br>--<br>Baseline 8S<br>vs<br>8S (day 21)	****p<0.0001
